# Supplementary material for: Experiences of Adults High in the Personality Trait Sensory Processing Sensitivity: A Qualitative Study
Source: J Clin Med. 2021 Oct 24;10(21):4912. doi: 10.3390/jcm10214912 (PMC8584340; doi:10.3390/jcm10214912)
Supplement: Supplementary file 1 [file jcm-10-04912-s001.zip › jcm-1415905-supplementary.pdf]

Table S1. Demographic characteristics of the eligible individuals, selected individuals, and the final sample, classified in groups used for the random stratified sampling.

Group	Eligible participants registering their interest: N	Selected participants: N	Interviewed participants: N	Reason for not participating
Men				
Lower educated[1]				
Younger (25-37)	3	3	2	Personal circumstances (1)
Older (38-50)	8	7	5	Personal circumstances (1); preference face-to-face (1)
Higher educated[1]				
Younger (25-37)	8	4	3	Personal circumstances (1)
Older (38-50)	17	5	3	No response to invitations (2)
Women				
Lower educated[1]				
Younger (25-37)	28	7	4	Personal circumstances (2); no response to invitations (1)
Older (38-50)	70	3	3	
Higher educated[1]				
Younger (25-37)	129	3	3	
Older (38-50)	170	3	3	
Total	433	35	26	

[1]Education level was dichotomised and a higher education level was defined as having completed a university or university of applied sciences education [22]. All lower and middle education levels, including pre-vocational secondary education, pre-university education, and secondary vocational education, were classified as a lower education level.

Table S2. COREQ (COnsolidated criteria for REporting Qualitative research) Checklist.

Topic	Item No.	Guide Questions/Description	Reported on Page No.
**Domain 1: Research team and reflexivity**			
Personal characteristics			
Interviewer/facilitator	1	Which author/s conducted the interview or focus group?	5
Credentials	2	What were the researcher's credentials? E.g. PhD, MD	5
Occupation	3	What was their occupation at the time of the study?	5
Gender	4	Was the researcher male or female?	5
Experience and training	5	What experience or training did the researcher have?	5
Relationship with participants			
Relationship established	6	Was a relationship established prior to study commencement?	5
Participant knowledge of the interviewer	7	What did the participants know about the researcher? e.g. personal goals, reasons for doing the research	5
Interviewer characteristics	8	What characteristics were reported about the interviewer/facilitator? e.g. Bias, assumptions, reasons and interests in the research topic	/
**Domain 2: Study design**			
Theoretical framework			
Methodological orientation and Theory	9	What methodological orientation was stated to underpin the study? e.g. grounded theory, discourse analysis, ethnography, phenomenology, content analysis	5, 6
Participant selection			
Sampling	10	How were participants selected? e.g. purposive, convenience, consecutive, snowball	3
Method of approach	11	How were participants approached? e.g. face-to-face, telephone, mail, email	5
Sample size	12	How many participants were in the study?	3, 4
Non-participation	13	How many people refused to participate or dropped out? Reasons?	3, 4 / Table S1
Setting			
Setting of data collection	14	Where was the data collected? e.g. home, clinic, workplace	5
Presence of non participants	15	Was anyone else present besides the participants and researchers?	/

Description of sample	16	What are the important characteristics of the sample? e.g. demographic data, date	6
Data collection			
Interview guide	17	Were questions, prompts, guides provided by the authors? Was it pilot tested?	5
Repeat interviews	18	Were repeat interviews carried out? If yes, how many?	N/A
Audio/visual recording	19	Did the research use audio or visual recording to collect the data?	4
Field notes	20	Were field notes made during and/or after the interview or focus group?	5
Duration	21	What was the duration of the interviews or focus group?	5
Data saturation	22	Was data saturation discussed?	6
Transcripts returned	23	Were transcripts returned to participants for comment and/or correction?	5
**Domain 3: analysis and findings**			
Data analysis			
Number of data coders	24	How many data coders coded the data?	5, 6
Description of the coding tree	25	Did authors provide a description of the coding tree?	/
Derivation of themes	26	Were themes identified in advance or derived from the data?	5
Software	27	What software, if applicable, was used to manage the data?	5
Participant checking	28	Did participants provide feedback on the findings?	5
Reporting			
Quotations presented	29	Were participant quotations presented to illustrate the themes/findings? Was each quotation identified? e.g. participant number	8 – 12 / Table S4 / Table S5
Data and findings consistent	30	Was there consistency between the data presented and the findings?	6 – 12
Clarity of major themes	31	Were major themes clearly presented in the findings?	6 – 12
Clarity of minor themes	32	Is there a description of diverse cases or discussion of minor themes?	6 – 12

Developed from: Tong A, Sainsbury P, Craig J. Consolidated criteria for reporting qualitative research (COREQ): a 32-item checklist for interviews and focus groups. *International Journal for Quality in Health Care*. 2007. Volume 19, Number 6: 349 – 357

Table S3. Means and standard deviations on HSP subscales.

Subscale	M (SD)
Ease of excitation	5.33 (0.65)
Low sensory threshold	5.22 (0.83)
Aesthetic sensitivity	5.80 (0.71)

Table S4. Identified SPS characteristics, including example quotes.

Theme	Subtheme	Example quotes
**Emotional responding**	Strong negative emotions	'When I am at a party for example and I say, like: "What nice tableware" or something like that and somebody says, like: "Well, I think it actually looks terrible", then I can suddenly start thinking: ouch! That it cuts me deeper than I perceive it cutting people around me. And then I really feel like a scared little animal that's easily hurt or something.' (p20, female, aged 25-37, lower educated) 'Two weeks ago I asked, I had promised my son to get a croissant from the McDrive. So we drove up there and there was a sign that it was completely blocked off, which meant we couldn't get to the drive-through. But I saw a man standing there washing the windows or something. So I went up to him and asked: "Gee, what's going on? Why can't we go in?" and he snarled at me "Can't you see that they're renovating here!" Well, he was really mad or something. He probably had a bad day, but that hits me really hard. And then I drove off, two kids in the car and I'm just crying because I think he's so angry at me.' (p23, female, aged 25-37, higher educated)
	Strong positive emotions	'Very small things, I can intensely enjoy those, yeah. Small things that people often overlook. A breeze through your hair for example, or the sun that shines, or leaves that make that rustling sound when you walk over them in autumn.' (p9, female, aged 38-50, lower educated)<br>'I walked in and this kind of flashmob was just starting when I entered, so apparently there were all these performances, and it appeared that there was a choir amongst the crowd. And suddenly they all stood up and started singing, beautifully. Well, I was standing there at the entrance, cyring, I thought it was so beautiful. Then I think: wow, what is happening here? That was so beautiful, yes.' (p11, female, aged 38-50, higher educated)
	Taking much time to process emotions	'It seems that many people don't mind it if they, for example, look at a video which contains violence. They can just scroll away. But with me it actually sticks. It hits me really hard and it just sticks.' (p22, male, aged 38-50, lower educated)<br>'When someone says something that hits me very hard and I discuss that with someone else, and they say: "Oh, but can't you just let that slide off of you?" I think: no, I can't!' (p20, female, aged 25-37, lower educated)
**Relatedness to others**	Noticing or feeling people's emotions	'I can just look around the group and immediately spot who is feeling well and who is fighting as a couple, or where there's tension.' (p10, male, aged 38-50, higher educated) "I get emotional very quicly when someone around me gets emotional." (p18, female, aged 38-50, higher educated)
	Attention to others	'When I work together with colleagues for example, I very quickly know like: this colleague likes this, this colleague likes that. So, I often get told that collaborating is nice, because I, of course, think like: oh, that one likes to do things that way, and that one likes to do

		things that way. But more often than not I forget to think about my own interests.' (p8, female, aged, 25-37, higher educated)<br>'It's always about others and never really about yourself. It's almost always me that organizes the family trips. Or at work, with the mechanics, if they want time off and it's not really possible, I still try to make it possible. Or when a customer calls because there's a malfunction, yeah, I always try to fix it immediately.' (p22, male, aged 38-50, lower educated)
	Understanding people's emotions and intentions	'When I first make contact with children, especially when they are new arrivals, I very quickly notice […] I start reacting very enthusiastically or I need to really stay calm. Yeah, for the most part I quickly realise how a child is put together and how I can respond to the situation.' (p14, male, aged 25-37, lower educated) 'I remember when I was in my twenties, I was sitting on the train and there was an eldery gentleman who had somehow not properly stamped his ticket or something and so he was faced with an angry conductor. And uhm, you just saw, you could see that that man really didn't do that on purpose, he was just confused, or he didn't understand it completely or something.' (p11, female, aged 38-50, higher educated)
	Acting on empathy	'When someone says, for example, like "sure, I'm okay", I might want to ask like "hey, how are you really? Because I see this and this."' (p21, male, aged 25-37, higher educated)<br>'The same friend that I had helped […] lost her daughter five years ago. Everyone dropped her, except me. I then take action and help. I'm thinking: you need help right now. And that, well, I think, why don't others see that? Why don't they realise that you shouldn't drop someone at that moment?' (p16, female, aged 38-50, lower educated)
	Feeling connected with others	'When I am really good friends with someone, that can be a real deep feeling you know? A true connection, as if it's family I guess. So, when something negative happens or there's a fight one time or something, then it kinda really hits me hard.' (p19, female, aged 25-37, lower educated)<br>'What quality would you really miss if you'd wake up tomorrow and no longer be highly sensitive?' ' […] The connection with everyone around you, with everything around you. Just being there and enjoying everything. I'm feeling gratitude for everything life has to offer, and especially those connections. I can simply sit on a bench and enjoy other people being all busy together.' (p27, male, aged 38-50, lower educated)
**Thinking**	Worrying and ruminating	'Like when I finally lie in bed and go to sleep, everything keeps like rolling around in my head and I keep mulling things over. And yes, I do mean every time, I can never get to sleep in one go. For example, my ex, they always said good night and they were gone within ten seconds, but I would keep thinking for an hour before I fell asleep.' (p14, male, aged 25-37, lower educated)<br>'I cared more about something, because when something had happened, I looked very much at myself, like, oh, that was my own fault. I should've done that, then it wouldn't have happened or I should have done that, then it would have turned out differently. And that part of me is, I think, very much related to being highly sensitive.' (p23, female, aged 25-37, higher educated)
	Thinking and reflecting a lot	'Sometimes it takes awfully long for me to make a decision, because I want to weigh all the options and the pros and cons and what everyone's opinion might be. These are mostly, for example, things we are going to do, and my girlfriend then thinks: get on with it already.' (p6, male, aged 38-50, higher educated) 'When you've had a nice evening with a friend, or nice or not so nice contact with a client, I can spend the whole afternoon thinking about

		it, if I've used the right words or if I might have hurt someone, or if I stood up for myself enough.' (p4, female, aged 25-37, lower educated)
	Need for depth and meaning	'My father always said: "you always get so philosophical right off the bat". And then I think: What are you talking about? I am just talking about the state of my life or how I experience something or think about it and he immediately thinks it's deep?' (p11, female, aged 38-50, higher educated)<br>'I do like to discuss but only in a positive way. I can do it whole evenings just for fun. Maybe it's because I'm looking for a certain challenge in it.' (p25, male, aged 38-50, lower educated)
**Overstimulation**	Overstimulation by sensory stimuli	'Well, at my work at [company] we naturally have rather bright lights to nicely illuminate all those [products], but sometimes I would just stand still for a moment […] but then if one of those bright lights might shine on my face in that particular spot, I would step aside, because I know it might cut really deep and would need to be processed, so to speak.' (p19, female, aged 25-37, lower educated)<br>'When everyone is very busy, I get just very tired of it, [I get tired] of sounds too. I also experience that in my living room. Sometimes, a sound or a bag of crisps or something really drives me crazy, and my daughter experiences that too.' (p6, male, aged 38-50, higher educated)
	Overstimulation by social stimuli	'I actually don't go to large events in large venues anymore. I do that very rarely because I know I can actually only take it for like an hour or something.' (p13, female, aged 38-50, higher educated)<br>'Crowded birthdays, all those stimuli and the hustle and bustle and people and keep being sociable, those are the elements that I'm still struggling with a bit.'<br>(p8, female, aged, 25-37, higher educated) 'I am self-employed, so there are no stimuli from colleagues […] which have affected me a lot in the past, when I still worked in an office.' (p4, female, aged 25-37, lower educated)
	Effects of overstimulation on cognition	'I actually have a lot of problems making decisions when I experience some overstimulation. Well, I have that after a day of work when I stand in the supermarket, where I think: I can't take this, I'll just make spaghetti bolognaise again or whatever.' (p5, male, aged 25-37, higher educated)<br>'I cannot work when the radio is on. At the beginning of Corona, me and my boyfriend both worked downstairs and the radio was on and my boyfriend was on the phone, well I could not do anything. I would look at him like how can you work with the radio on? Well, it is very normal for him, he is absolutely not highly sensitive. So I really need to have silence, else I cannot focus. That is really awful.' (p18, female, aged 25-37, higher educated)
	Effects of overstimulation on mood	'People who are constantly clicking their pens, oh, please stop. I can't stand it. I get incredibly annoyed. I don't know what it does to me specifically. I'm not that far yet. But I get incredibly annoyed by it. I just go off like a firework, I just get angry and rebellious and then I'm like: cut that out.' (p16, female, aged 38-50, lower educated)<br>'When I have a busy day, then I am just really irritable. I also hear from others that I have mood swings and that can all be traced back to when I have been in a busy environment, or been in a restaurant, or, or a pub. That is even worse. At a certain point I just cannot handle it anymore because of all the stimuli that affect me, and because of that my mood, yes, will become less positive.' (p12, male, aged 38-50, higher educated)

**Perceiving details**	Perceiving a greater quantity of information	'I sometimes specifically notice that when you walk past some place while you're with someone, you might see something really beautiful and the person you're with just completely misses it or walks past it.' (p24, male, aged 38-50, higher educated)<br>'How would you describe a highly sensitive person?' 'Yes, just someone who is more affected by stimuli and needs to put in more effort to filter them.' (p28, female, aged 25-37, lower educated)
	Perceiving information in greater detail	'I absolutely think perceiving the world in a sort of high definition' (p26, female, aged 25-37, higher educated)<br>'You notice a lot more details of things. For example, my wife showed me a picture this morning, a baby picture, never seen it before. "Who is that?", she asks. I say: "that's your father". But I've only known her father since he was a grownup. She asked: "I hadn't even recognised him. How did you know?" "Well, his eyes, his ears, I guess... I dunno."' (p9, female, aged 38-50, lower educated)<br>'For example, you are in a group and people have a certain facial expression for a short moment. Those kind of things stand out. For example, you notice the facial expressions when you enter the room and it already gives you some information. After that, they, for example, have a different face that they put on. Those differences, that can be positive and negative, stand out more easily.'<br>'What exactly are you looking at when you look at someone's facial expressions?' 'The eyes, the mouth, frowning, maybe tension in somebody's cheeks, those kinds of things. If the lips are tense, all those kind of things.' (p17, male, aged 25-37, higher educated)
**Global SPS characteristics**	Self and identity	'It [HSP] really encompasses your entire being.' (p4, female, aged 25-37, lower educated)<br>'I had many problems with being the way I am, which is of course also caused by high sensitivity.' (p16, female, aged 38-50, lower educated)<br>'I noticed that I approached things differently, that I looked at things differently and that I absorbed information differently and when something happened, I needed time to process things my way.' (p27, male, aged 38-50, lower educated)
	Feeling stressed	'I would like to be able to relax more, that is, in the evenings. I am constantly in 'hurry-up' mode.' (p7, male, aged 38-50, lower educated)<br>'I find it difficult when much of me is expected, I can easily feel pressured.' (p18, female, aged 38-50, higher educated)<br>'I found out that I find it difficult to perform under time pressure.' (p15, male, aged 25-37, lower educated)
	Feeling tired	'It's as if I'm more conscious of those details, those expressions, of what comes back to me. And it's a lot of fun and actually quite useful. But it can also be quite tiring without you noticing it.' (p25, male, aged 38-50, lower educated)<br>'For instance, when it is really crowded, or when there is so much noise, I can stay there, for a few hours, but that really takes a lot of effort. And then I am just a bit total loss.' (p6, male, aged 38-50, higher educated)

Table S5. Identified strategies and activities to improve well-being, including example quotes.

Theme	Subtheme	Example quotes
Reducing sensory input	Solitude and quiet environments	'Look, at my work I try to walk a little round at my breaks, you know, on my own. People do not understand that either, like: why do you not want to walk with us? That is really a moment for myself. Yes, a lot of people do not understand that, but you try to be alone for a little while and, yes, I can really enjoy, for example, a tree that I encounter on the way or the sun, or who knows what.' (p22, male, aged 38-50, lower educated)
		'I did experience that as pleasant [due to the Corona situation], because I had more time to withdraw and then I could process the stimuli better. So maybe in that, Corona, the lockdown or staying indoors was a positive experience for me.' (p20, female, aged 25-37, lower educated)
	Reducing specific sensory input	*'How would you describe a highly sensitive person?'* 'Someone who […] always wears sunglasses in the car.' (p12, male, aged 38-50, higher educated)
		'For example, when I know that it will be a busy day and I have to go by train, I just use earplugs.' (p26, female, aged 25-37, higher educated)
Psychological strategies	Support from others	'Friends of mine, who experience the same, yes, I really like that we can talk about it sometimes, because you have similar experiences. It's not often a topic of conversation, but sometimes it is, or when someone expresses she does not feel like doing something and you can say oh yes, I can experience the same thing. Yes, the recognition we find with each other is nice.' (p18, female, aged 38-50, higher educated)
		'[someone who] does not find it a problem when I just want to leave a place, where there is too much noise, too much light or too many people. That you do not mind when I say: "Yes, I just do not want to be here anymore. So, shall we leave?" That is what I think.' (p19, female, aged 25-37, lower educated)
	Mindfulness, yoga, and meditation	'After dinner, [I] go upstairs for a little while, take your moment, just half an hour and I will lay down and meditate and then I think, ok, I am back.' (p3, female, aged 38-50, lower educated)
		'Meditation, is really super hip. But it really helps me, so I just try to make it a daily activity. It is not easy, but I try.' (p11, female, aged 38-50, higher educated)
	Positivity, acceptance, and reflection	'At times when you just do not feel very well, then accept okay, maybe today will be a less productive day, or a less pleasant, happy day. To accept that and, well, just, go with the flow and see what happens.' (p21, male, aged 25-37, higher educated)
		'Before, things happened that could leave me completely shattered and I did not understand that. And now I learned that when something like this happens to me, that I just need to spend an hour on: where is it located? Does this belong to me? Does this belong to somebody else? How important is this?' (p16, female, aged 38-50, lower educated)
